# Human-Based Immune Responsive In Vitro Infection Models for Validation of Novel TLR4 Antagonists Identified by Computational Discovery

**DOI:** 10.3390/microorganisms10020243

**Published:** 2022-01-22

**Authors:** Helena Merk, Tehila Amran-Gealia, Doris Finkelmeier, Christina Kohl, Isabelle Pichota, Noa Stern, Steffen Rupp, Amiram Goldblum, Anke Burger-Kentischer

**Affiliations:** 1 Institute of Interfacial Process Engineering and Plasma Technology, University of Stuttgart, Nobelstr. 12, 70569 Stuttgart, Germany; helena.merk@gmx.de; 2 Laboratory of Molecular Modelling, Faculty of Medicine, Institute for Drug Research, The Hebrew University of Jerusalem, Jerusalem 91120, Israel; tehila.gealia@gmail.com (T.A.-G.); noastern10@gmail.com (N.S.); 3 Fraunhofer Institute of Interfacial Engineering and Biotechnology IGB, Nobelstr. 12, 70569 Stuttgart, Germany; doris.finkelmeier@igb.fraunhofer.de (D.F.); christina.kohl@igb.fraunhofer.de (C.K.); i.pichota@web.de (I.P.)

**Keywords:** 3D-immune-competent Infection models, *Candida albicans*, Toll-like receptors, antagonist, pharmacophore, docking

## Abstract

Infectious diseases are still a major problem worldwide. This includes microbial infections, with a constant increase in resistance to the current anti-infectives employed. Toll-like receptors (TLRs) perform a fundamental role in pathogen recognition and activation of the innate immune response. Promising new approaches to combat infections and inflammatory diseases involve modulation of the host immune system via TLR4. TLR4 and its co-receptors MD2 and CD14 are required for immune response to fungal and bacterial infection by recognition of microbial cell wall components, making it a prime target for drug development. To evaluate the efficacy of anti-infective compounds early on, we have developed a series of human-based immune responsive infection models, including immune responsive 3D-skin infection models for modeling fungal infections. By using computational methods: pharmacophore modeling and molecular docking, we identified a set of 46 potential modulators of TLR4, which were screened in several tests systems of increasing complexity, including immune responsive 3D-skin infection models. We could show a strong suppression of cytokine and chemokine response induced by lipopolysacharide (LPS) and *Candida albicans* for individual compounds. The development of human-based immune responsive assays provides a more accurate and reliable basis for development of new anti-inflammatory or immune-modulating drugs.

## 1. Introduction

Infectious diseases are still a major problem for humankind, both in the industrialized and developing world. This includes microbial infections, both bacterial and fungal infection, with a constant increase in resistance to the current anti-infectives used [1]. Innate immunity is one of the first lines of immune defense, preventing pathogen invasion. Therefore, modulating innate immunity has been suggested as a promising approach to support anti-infective therapy. Pattern recognition receptors (PRRs) play a central role in regulating the innate immune system [2,3]. They are ubiquitous in nature and found in plants, insects, vertebrates, and in humans. Today, PRRs are recognized as receptors with many functions, including the sensing of invading pathogens by recognition of pathogen-associated molecular patterns (PAMP), as well as endogenous danger signals from one’s own cells, shaping the gut microbiome, involvement in cancer and autoimmune disease, and even non-immunological functions [4,5,6,7,8]. The Toll-like receptors (TLR) are among the most well studied immune-receptors in humans. They are considered drug targets for several indications, from anti-infectives to tumor therapeutics [9]. TLR4 and its co-receptors, MD2 (myeloid differentiation factor 2, also called Lymphocyte antigen 96) and CD14 (cluster of differentiation 14) are especially interesting, having an important role in host immune response to pathogens and in many acute and chronic inflammatory diseases, including rheumatoid arthritis and cancer. They are crucial for recognizing fungal and bacterial pathogens, via O-Mannan and lipopolysaccharide (LPS), respectively, and are implicated in septic shock [10,11,12]. Therefore, TLR4 and its co-receptors are promising drug targets for therapeutics in sepsis and in concomitant inflammatory processes [6]. Some of the therapeutic TLR modulators are derived from natural ligands, other molecules lacking obvious similarities to the natural ligand have also been identified as TLR modulators and are intensively examined for their potential as therapeutics [13,14]. Moreover, TLR agonists are continuously developed as candidate adjuvants for vaccination, with several products already in the market [15,16]. However, multiple clinical trials on TLR-modulators have been terminated at different stages, due to efficacy and safety issues [9]. Therefore, a major challenge is to validate efficacy and safety as early as possible in drug development, especially since animal models often do not reflect human immune response. Thus, both more reliable preclinical test systems and new TLR modulators are needed.

We have recently published an immune-competent infection model of the human skin, which was used to mimic *Candida albicans* infections [17,18]. This infection model revealed that in addition to keratinocytes and immune cells, dermal fibroblasts play a critical role in antimicrobial defense. Triggering the fibroblasts antimicrobial response via expression of antimicrobial peptides such as β-defensins and LL-37 required the presence of PBMCs or CD4+ T cells in the infection model [18]. In this study, we employ this infection model together with other, less complex ex vivo/in vitro models to validate TLR4 modulators identified using a computational approach. This approach follows a similar strategy as described by us previously, using computational discovery approaches and experimental confirmation of the molecules selected in cell-based assays [14,19]. To identify novel ligands for the TLR4/MD2 co-receptors, we employed in this study both ligand and structure-based pharmacophore as the main computational method for modeling and subsequent screening of novel TLR4/MD2 modulators. This procedure resulted in a set of 46 potential modulators. The specific binding of these potential ligands to TLR4/MD2 and their effect was examined using in a first step a set of cell-based assays and in a second step the more complex human-based immune responsive 3D-infection models. We found a strong antagonistic activity of a small molecule (T6030504, 3-[(2-methoxyphenyl)formamido]-N-(5-methylpyridin-2-yl)propanamide) that efficiently blocks TLR4-mediated response to *C. albicans* as well as to LPS in several immune-responsive models. These findings are the starting point for further development of the compound identified into a novel anti-inflammatory drug, which could be employed to moderate overshooting reactions of the immune system, such as sepsis or dampening autoimmune diseases such as for example arthritis. More elaborate human-based disease models for specific indications are key for further development.

## 2. Materials and Methods

### 2.1. Computational Methods

The main computational method employed for modeling the TLR4/MD2 interaction with small molecules and for subsequent screening, is the 3D pharmacophore method [20]. Two approaches were employed for developing pharmacophore models: “ligand based” and “structure based”. The “ligand based” pharmacophore uses a set of ligands that is known to interact with the same biological target, in order to identify (partially by conformational analysis) which ligand features are common to that set. It is best to compare active and highly active ligands to ligands that have weak or no interaction with that target in order to have a better validation of the features. “Structure based” pharmacophore is based on crystal structures of complexes between ligands and biological targets, and even a single such structure may supply the required features of ligand–protein interactions. A few solved complexes of a protein with different ligands, as frequently available in the Protein Data Bank (PDB) (http://rcsb.org, accessed on 6 August 2013) increase the chances for a better-defined pharmacophore. The protein is used only for determining the interaction features between the ligand and the protein. Once these features are determined, both protein and ligand (or ligands) are not required for the subsequent screening. The LigandScout software [20] (INte: Ligand, Maria Enzersdorf, Austria) was used for the detection and interpretation of crucial interaction patterns in both, TLR4/MD2 ligands and the crystal structures of TLR4/MD2 complexes. As we wish to discover novel inhibitor modifiers of TLR4/MD2, we picked the structure of the PDB complex with Eritoran, structure code 2Z65 [21] which has a better resolution (2.7 Å) than that of 3ULA (3.6 Å) [22], another complex with Eritoran. After pharmacophore construction and screening, docking to the 2Z65 structure was used as a final step for picking candidate molecules that were detected by the pharmacophore method. Docking was performed by two different programs: FlexX in LeadIT [23] (BioSolveIT, Sankt Augustin, Germany) and FRED [24] (OpenEye Scientific Software, Santa Fe, NM, USA).

Another approach for discovery was to use “ligand based” pharmacophore models. Those were constructed based on antagonists from the European Molecular Biology Lab website ChEMBL [25] with a molecular weight lower than 500 Dalton, containing 18 inhibitors of TLR4/MD2 with variable experimental IC_50_ values, which were used to construct ligand-based pharmacophore models. 

For screening, we used the Enamine “hit finding” catalog (https://enamine.net/hit-finding, accessed on 15 January 2014) of ~1.8 million molecules (at the time, now over 2.5 million for immediate delivery). Screening by the “ligand based” pharmacophore provided 16 molecules while combining “ligand based” with “structure based” and docking provided 30 more molecules. Overall, 46 molecules were identified for in vitro testing.

### 2.2. Preparation of Immune-Modulatory Compound

A larger amount of the compound (T6030504, CAS 1007791-58-3, C_17_H_19_N_3_O_3_, IUPAC Name 3-[(2-methoxyphenyl)formamido]-N-(5-methylpyridin-2-yl)propanamide) used for assays with immune cells and skin infection-models was synthesized by Sohena GmbH, Tübingen, Germany, with 98% purity determined with HPLC analysis. It was diluted in 100% DMSO (Carl Roth GmbH, Karlsruhe, Germany) to reach full solubility. Compound T6432438 and all other compounds used for screening were purchased from Enamine, Kiev, Ukraine or from EMC microcollections, Tübingen, Germany. For cell culture experiments, the samples were diluted in cell culture media to a final DMSO concentration of 0.2%.

### 2.3. In Vitro Cell-Based Reporter Gene Assay

To validate the immune-modulating compounds selected by computational methods, the previously described reporter cell lines NIH 3T3 TLR4/CD14 and NIH 3T3 TLR4/MD2 were used. The setup of the assay was described previously [19,26]. In brief, the cells were cultivated in a T-75 cm^2^ flask until confluence of 90% and were then distributed in a 96-well plate with a cell number of 2.5 × 10^5^ cells/well. The cultivation media (DMEM high glucose (4.5 g/L), 10% FCS, 100 U/mL penicillin, 100 μg/mL streptomycin, 2 mM l-glutamine; Invitrogen) were exchanged before performing the cell-based reporter gene assay (PAMP-assay) (DMEM high glucose (4.5 g/L), 0.5% FCS, 100 U/mL penicillin, 100 μg/mL streptomycin, 2 mM l-glutamine) [19]. LPS (WHO 3rd International Standard, NIBSC code: 10/178, E. coli 0113: H10: K-endotoxin), known as one of the main activating TLR4 receptor ligands (agonist) was used at a concentration of 25 pg/mL (0.2 EU/mL) for TLR4/CD14 cells and 25 ng/mL (208 EU/mL) for TLR4/MD2 cells. To monitor the NF-κB-dependent secreted alkaline phosphatase (SEAP) expression, the SEAP-substrate para-Nitrophenylphosphate (p-NPP, Merck, Darmstadt, Germany) was used. The substrate (50 µL) was added to 50 µL taken from the supernatant of the reporter cell culture after incubation for 16 h unless otherwise noted.

### 2.4. Cytotoxicity Assay/Neutral Red Assay

The TLR4 modulators were assessed for cytotoxic effects on NIH 3T3 cells and human primary fibroblasts with neutral red. Neutral red-assay is based on the absorption of the dye neutral red (Merck, Darmstadt, Germany) into the lysosomes of vital cells. The dye was dissolved by ethanol:water:acetic acid (50:50:1%) solution and cellular uptake was measured at a wavelength of 540 nm.

### 2.5. Isolation and Application of Peripheral Blood Mononuclear Cells

Human buffy coats were obtained from healthy donors at the blood donation center Katharinenhospital, Stuttgart, Germany and from male volunteers of age 25–35 years. For peripheral blood mononuclear cells (PBMCs) isolation, the buffy coat was diluted 1:2 in 0.9% NaCl (Carl Roth, Karlsruhe, Germany) and transferred to leucosep tubes (Greiner Bio-one, Frickenhausen, Germany), containing separation medium and a porous barrier. After centrifugation at 800× *g* for 15 min at room temperature with the brake off, the PBMCs layer was collected and washed twice in PBS (Thermo Fisher Scientific, Darmstadt, Germany). Residual erythrocytes were removed by lysis with NaCl solution: 3 mL of 0.2% and 1.6% for 30 s, respectively.

For the experiments, PBMCs (4 × 10^6^/mL) were transferred to Eppendorf tubes in RPMI medium (Thermo Fisher Scientific, Darmstadt, Germany) supplemented with 2% heat-inactivated FCS, 1% l-glutamine (200 mM, (Thermo Fisher Scientific, Darmstadt, Germany) to final concentration of 2 mM), and 1% penicillin-streptomycin (PenStrep 100× (10.000 U/mL), Thermo Fisher Scientific, Darmstadt, Germany, to 100 U/mL penicillin and 100 μg/mL streptomycin). For stimulating TLR4, 200 pg/mL LPS (3rd endotoxin WHO standard, NIBSC, London, UK) was used. Several concentrations of antagonist T6030504 were used, as indicated, to block LPS stimulation. The PBMCs with LPS and/or T6030504 were incubated for 24 h at 37 °C. After centrifugation at 350× *g* for 10 min, the levels of the cytokine IL-1β in the culture supernatants (200 µL/tube) were measured by using Human IL-1β Platinum ELISA Kit (Thermo Fisher Scientific, Darmstadt, Germany). For viability testing, the cells were resuspended in diluted neutral red solution (Merck, Darmstadt, Germany, 1:50 diluted in RPMI supplemented with 2% FCS), and incubated for 3 h at 37 °C. The cells were washed once in RPMI supplemented with 2% FCS and lysed with a solution of water, ethanol, and acetic acid (50%:50%:1%). After plating the lysed cells into 96-well plates, the absorption at 540 nm was measured with a spectrophotometer (Spectramax plus 384, Molecular Devices, LLC, Ismaning, Germany).

### 2.6. Human Whole Blood Assay

Human whole blood was obtained from female volunteers at the age of 30 years using a Litium/Heparin Monovette (Sarstedt, Nümbrecht, Germany). The samples were diluted 1:11 in RPMI media and incubated for 24 h in a humidified incubator at 37 °C, 5% CO_2_. LPS (WHO 3rd International Standard, NIBSC code: 10/178, *Escherichia coli* 0113: H10: K–endotoxin, 0.8 EU/mL) was used as TLR4-specific ligand, known to induce IL-1β in human immune cells. The supernatant was collected by centrifugation and used for further ELISA measurements using abcam46052 Kit (Abcam, Berlin, Germany).

### 2.7. Antagonist Activity in an In Vitro Co-Culture Infection Model

The co-culture in vitro assay consists of human primary fibroblasts (50,000 cells/well of a 24 well plate; Greiner Bio-one, Frickenhausen, Germany) and PBMCs (1 × 10^6^ cells/well) in a corresponding trans-well insert with a pore size of 3 µm. The fibroblasts were isolated from human biopsies of the skin as described in [27]. PBMCs were isolated from voluntary male donors as described above. The confluent cell layer of fibroblasts was infected with *C. albicans* (100 CFU/well, clinical isolate SC5314) grown overnight in yeast peptone dextrose broth (YPD, BD, Heidelberg, Germany) at 30 °C and 350× *g* and incubated for 48 h. The TLR4 antagonist was added 6 h before infection with *C. albicans* to the immune cells at a concentration of 50 µM. The cytokine response was measured with the ELISA kit abcam46052 (Abcam, Berlin, Germany) for IL-1β and abcam83700 for CXCL-10 using 200 µL of the media supernatant. 

### 2.8. Complex In Vitro Skin Infection Model

Skin models were generated as described previously [17,18]. SF1-fibroblasts [28] were embedded in a collagen matrix for the dermal part, Ker-CT-cells (ATCC-CRL4048) were grown on top as the epidermal part and CD4-positive T cells or PBMCs were added below the dermal part of the skin. The immune cells were isolated from voluntary male donors. Histopaque gradient (Merck, Darmstadt, Germany) was used to separate the PBMCs from erythrocytes and granulocytes. The PBMC cell layer was used for further isolation of CD4^+^ T cells with antibody separation using the naïve CD4^+^ T-cell Isolation Kit II (Miltenyi Biotech, Bergisch Gladbach, Germany) and activated with Dynabeads^®^ Human T-Activator (Thermo Fisher Scientific, Darmstadt, Germany) with CD3/CD28-labeled beads for 24 h at a cell-to-bead ratio of 5:1. The immune cells were pre-incubated for 6 h with the antagonist before integration in the skin model. After 24 h the skin model was infected with 100 CFU of a *C. albicans* overnight culture (clinical isolate SC5314, 30 °C, 350× *g* in YPD (BD, Heidelberg, Germany) by adding the microorganism on top of the cornified epidermis for 48 h in a humidified incubator at 37 °C. After 48 h the skin models were fixed with Bouins solution (Carl Roth, Karlsruhe, Germany) for 1 h, and dehydrated and embedded in paraffin. The sections were cut in 4 µm tissue sections and de-paraffinized for Periodic acid-Schiff (PAS) staining. Staining of the fungi was performed with 1% periodic acid for 15 min and Schiff reagent (Merck, Darmstadt, Germany) for 30 min. Sections were washed three times with 0.05 M HCl, 0.6% Na_2_S_2_O_5_ and differentiated in tap water for 15 min. Afterwards tissue sections were stained with Mayer’s Hemalaun (Dako, Jena, Germany) solution (8 min) and Eosin B (Merck, Darmstadt Germany) (1 min). Hematoxylin is used to stain the cell nucleus and Eosin to monitor the formation of keratin in the epidermis. The dermal invasion of *C. albicans* was quantified by measuring the infected dermal area and the total dermal area using ImageJ (2012, Schneider).

## 3. Results

### 3.1. Computational Discovery of TLR4 Modulators

#### 3.1.1. Pharmacophore Modeling 

To construct a purely “ligand based” pharmacophore, we used 18 ligands (out of 25 TLR4 antagonists with IC_50_ values between 3.2–1000 nM and molecular weight up to 500 Da from the ChEMBL website). Then, a set of 1000 molecules were randomly picked from Enamine catalog by using the limitations of an “applicability domain”, so that the random molecules will be in the same chemical space as that of the active molecules. That is achieved by requiring that some of the properties will be close to those of the active molecules, that is, in the range of the actives’ average +/− 2 standard deviations for: No. of H-acceptors and H-donors, calculated logP and molecular weight. LigandScout software constructed three models from the active and the random molecules. By screening active molecules against randomly picked ones for the modeling, 2 out of 10 from the actives had a score above 0.8, while only 6 out of 1000 random molecules got such high scores. This model was subsequently used for virtual screening. 

Next, virtual screening through the pharmacophore models was performed: First, 360,000 molecules were picked from the Enamine database of 1.8 million molecules, having 4 or less rotatable bonds in order to reduce the loss of entropy upon binding and not to allow long molecules. By the applicability domain criteria mentioned above, they were reduced to 120,000 and were filtered by the pharmacophore models and by docking. About 1000 molecules passed the pharmacophore criteria to the docking stage. Most of these molecules were too small for interacting with enough of the pharmacophore features, and therefore we decided to include longer molecules, with 7–15 rotatable bonds. We found about 880,000 molecules (out of the 1.8 million from Enamine), which were reduced by applicability domain criteria to 260,000. About 2000 of those molecules passed the pharmacophore criteria to the docking stage.

**Figure 1:**
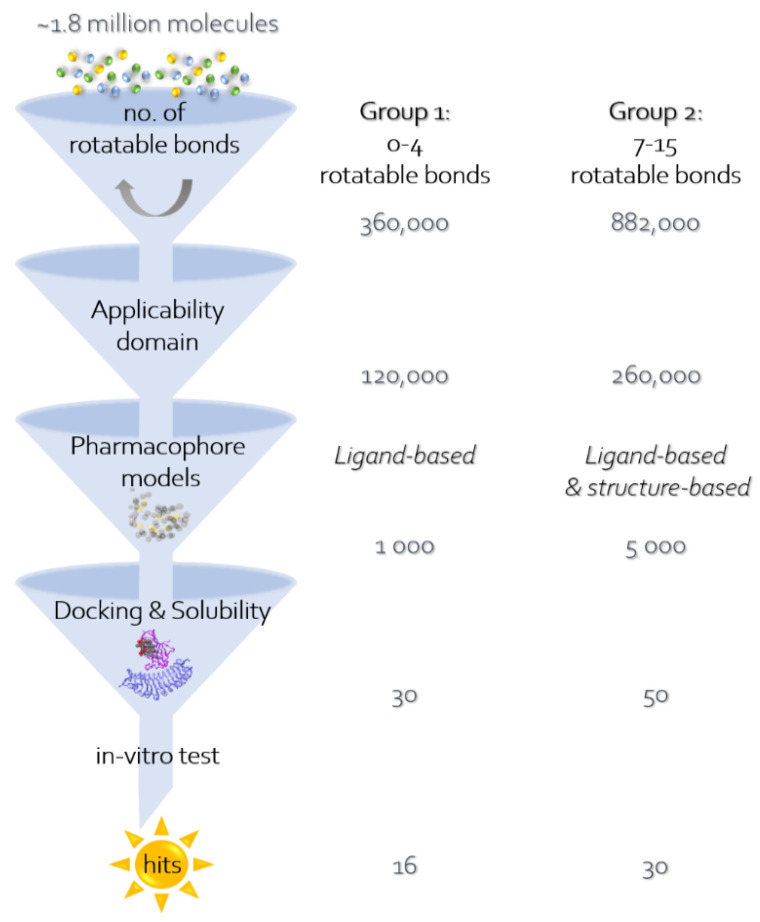
The computational procedure in this work includes the use of several methods or filters, one after another, in order to filter a large molecular database, Enamine, and find active compounds for the selected target, TLR4/MD2. (1) The first step is filtering by the number of rotatable bonds. There were 2 parallel filtrations: for molecules with 0–4 rotatable bonds (“group 1”) and molecules with 7–15 rotatable bonds (“group 2”). (2) The second step is filtering the resulting molecules of groups 1 and 2 from the previous step, by the applicability domain properties of the known active molecules: no. of donor atoms, no. of acceptor atoms, logP, and molecular weight. (3) The third step is filtering groups 1 and 2 from the previous step, with a pharmacophore model, which can be based on known ligands for the target or on a known crystal structure of the target. Specifically, group 1 was filtered only by a ligand-based pharmacophore model, while group 2 was filtered by a ligand-based, as well as a structure-based pharmacophore model. (4) The last step is filtering groups 1 and 2 from the previous step, by docking to TLR4/MD2 crystal structure (PDB code: 2Z65), using two docking programs: FRED and FlexX. The resulting molecules were then filtered by calculating solubility descriptors using three methods. The candidates that passed all of these filters were later examined in vitro to find whether they are indeed active on TLR4.

In addition to the ligand-based pharmacophore, the applicability-domain-filtered molecules were filtered by a structure-based pharmacophore model. The LigandScout software was used in this case as well. The pharmacophore model was based on the structure of Eritoran inside MD-2 (PDB code: 2Z65). Out of 260,000 molecules, 3000 were obtained in this filtration and added to the 2000 molecules obtained by the ligand-based pharmacophore model, and continued to the docking stage (Figure 1).

#### 3.1.2. Docking

By examining the interaction patterns in the crystal structures of TLR4/MD2 with Eritoran (PDB entries 2Z65 and 3ULA) and with LPS (PDB entries 3FXI and 4G8A) we found twenty-three significantly interacting amino acids in the MD2 pocket. Eight of these residues interact via H-bonds with the ligands, in most structures, and two prominent ones, Ser-120 and Tyr-102 present H-bonds in four structures and two structures, respectively. For virtual screening (VS) by docking, we require the docked ligand to interact with part of those residues.

**Figure 2:**
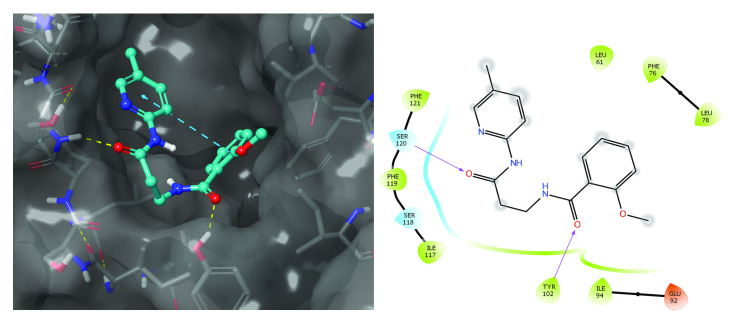
Compound T6030504 docked in TLR4-hybrid MD-2 pocket. On the left: T6030504 is shown in its docking position inside the MD-2 pocket. On the right: Schematic presentation of the interactions in that docking pocket. Most interactions are similar to those of the 2Z65 PDB structure of the MD-2 pocket with Eritoran. The two main H-bond donors to carbonyl oxygens of T6030504 are Tyr-102 and Ser-120 through their side-chain –OH, and Van der Waals (VdW) interactions with Phe-76, ILE-94, Ile-117, and Phe-119 were mentioned in Section 3.1.2. Ile-117, Ser-118, and Phe-121 are also part of the MD-2 pocket interactions with Eritoran. T6030504 has eight rotatable bonds, which include the NH-CO bonds. On the right: The closest VdW interactions of MD-2 residues to T6030504 are displayed as either continuous surface or isolated protein residues. Colors displayed are—light green for hydrophobic residues, light blue for polar residues, and brown for negatively charged.

Both sets that were based on a different number of rotatable bonds were docked to the 2Z65 structure and evaluated for their solubility by several algorithms. Among the top 100 molecules, about 60 had H-bonds with either Ser-120 or Tyr-102. Most of the other interacting residues are hydrophobic: Ile-63, Ile-74, Phe-76, Ile-94, Phe-104, Ile-117, and Phe-119. Finally, 46 molecules (30 with 7–15 single bond rotations and 16 with 0–4 rotations) were selected for experimental confirmation. Figure 1 gives an overview of the selection process and Figure 2 presents the docking position of T6030504 that was among the best performing molecules in the cell-based assay.

### 3.2. Experimental Validation of Computationally Selected TLR Modulators

#### 3.2.1. Cell-Based Assays to Confirm TLR4 Modulators

Experimental confirmation of the activity of the computationally selected molecules was performed using *C. albicans* as opportunistic fungal pathogen and LPS as strongly TLR4-activating ligand, part of the cell wall of Gram-negative bacteria, using a series of assays of increasing complexity. This ranged from cell-based reporter assays suitable for screening a larger set of molecules to 3D-immune responsive tissue models. 

**Figure 3:**
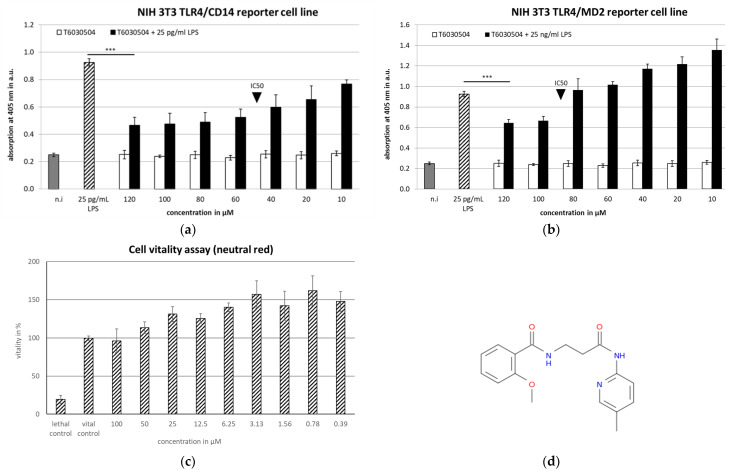
Cell-based screening assays for validation of TLR4 Antagonists: Dose-response analysis of T6030504 to identify the minimal inhibitory concentration necessary to block the TLR4 receptor complex in NIH 3T3 TLR4/CD14 (**a**) and NIH 3T3 TLR4/MD2 reporter cell lines (**b**). Black triangle points to the IC_50_-value of the compound. LPS was used to activate the TLR4 receptor complex in NIH 3T3 TLR4/CD14 reporter cells with a concentration of 25 pg/mL and in NIH 3T3 TLR4/MD2 reporter cells with a concentration of 25 ng/mL. The supernatant was collected and the substrate p-NPP was added to monitor the NF-κB-dependent SEAP secretion. The results are given for one representative experiment with three replicates. Significant differences are indicated with the *t*-test and *p* < 0.001 *** (**c**) Neutral red assay to detect cytotoxicity of the compound under test conditions with NIH 3T3 TLR4/CD14 cell line. The results are expressed as means ± SD for three experiments. (**d**) Chemical structure of T6030504.

Cell-based assays, specific for either TLR4/CD14 or TLR4/MD2 were employed to validate the 46 selected, commercially available compounds. The reporter-cells constitutively express the TLR4 receptor and either CD14 or MD2, respectively and contain a secreted alkaline phosphatase (SEAP) acting as a reporter, regulated by NF-κB [26]. We analyzed both, agonistic and antagonistic activity of the compounds. To detect the blocking activity of an antagonist, cells are treated with both the compound and with LPS from Gram-negative bacteria (see Table S1). LPS initiates the NF-κB-dependent pathway via TLR4 in the reporter-cell unless the compound applied blocks the receptor. Activating molecules are identified by directly adding the compounds to the reporter cells (see also [14,19]. The cell-based screening assays in this study identified seven compounds out of 46 high potential molecules with antagonistic activity against TLR4/MD2 close to or below 200 µM (Table S1 and Figure S1a–f and Figure 3). The two most active compounds, T6030504 and T6432438, showed activities in the lower µM range against both TLR4/CD14 and TLR4/MD2. The IC_50_ values of both compounds were determined using a concentration series as shown in Figure 3, for T6030504 with TLR4/CD14 (Figure 3a) and TLR4/MD2 (Figure 3b). For both cell-based assays, the IC_50_ was 50 µM (data for T6432438, TLR4/MD2-assay in Table S1, Figure S1f). Toxicity was evaluated using the neutral red assay. No toxic effect was observed on the reporter cells in the concentration range applied (Figure 3c). Both, T6030504 and T6432438, were further analyzed using several other cell-based assays specifically addressing additional TLRs, including TLR7/8/9 and TLR1/2/5/6. In none of these assays any effect on TLR-signaling was observed (not shown), indicating specific activity against TLR4/CD14 and TLR4/MD2. Due to better availability, we focused on one of the compounds, T6030504 (3-[(2-methoxyphenyl) formamido]-N-(5-methylpyridin-2-yl) propanamide) for further studies in more complex test systems using primary immune cells, whole blood, and immune-responsive 3D-infection models.

#### 3.2.2. T6030504 Blocks Cytokine Response in Human Whole Blood

**Figure 4:**
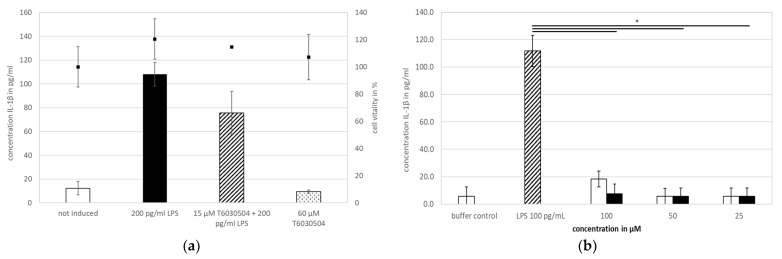
Characterization of T6030504 with primary human immune cells. Antagonistic activity is shown in PBMCs (buffy coat from multiple donors) and in human whole blood. (**a**) Effect of T6030504 on IL-1β secretion. PBMCs were incubated with 200 pg/mL LPS as TLR4 agonist and 15 µM T6030504 TLR4 antagonist. As control 60 µM T6030504 was added without LPS, showing no effect by itself. Black squares show the viability test via staining with neutral red, done in parallel. Data shown are given for one representative duplicate and expressed as mean ± SD. (**b**) IL-1β cytokine levels in the supernatant of human whole blood from donor 1999, A+. Whole blood is incubated with 100 pg/mL LPS in the absence and presence of the TLR4 antagonist (T6030504) at the concentrations indicated. Cytokine levels were measured in the supernatant after 24 h of incubation. The results are given for one representative duplicate experiment and expressed as means ± SD. Significant differences are indicated with the t-test and *p* < 0.05 *.

In the next step, we verified if T6030504 has an impact on primary human immune cells. Therefore, we analyzed the effect of the newly identified TLR4 antagonist on the cytokine response in human peripheral blood mononuclear cells (PBMCs) and in human whole blood in the presence of LPS as activating molecule, mimicking bacterial infection. The PBMC assay was performed using buffy coat from the blood donation center (multiple donors), whole human blood was derived from one single donor. The results are shown in Figure 4. As expected, the addition of LPS results in a strong IL-1β response of both, the isolated PBMCs (Figure 4a) and the immune cells present in whole human blood (Figure 4b). Addition of T6030504 to PBMCs isolated from buffy coat resulted in a reduction of the immune response induced by LPS (Figure 4a), however, without statistical significance. Cytotoxicity was assayed in parallel using the neutral red assay. No toxicity could be observed in the presence or absence of LPS (Figure 4a). In whole blood from a single donor (via Lithium-Heparin Monovette), the addition of T6030504 at all tested concentrations (15–60 μM) showed strongly reduced cytokine levels in the presence of LPS (Figure 4b). At a concentration of 100 μM the antagonist by itself induces a minor IL-1β response, indicating a potential stress response due to the addition of the compound. These results show that T6030504 is able to reduce the cytokine response of human immune cells to LPS in whole human blood significantly. In addition, this experiment shows that the compound is both stable and available for interaction with immune cells in whole blood.

#### 3.2.3. T6030504 Blocks Immune Response in an In Vitro Co-Culture Infection Model

**Figure 5:**
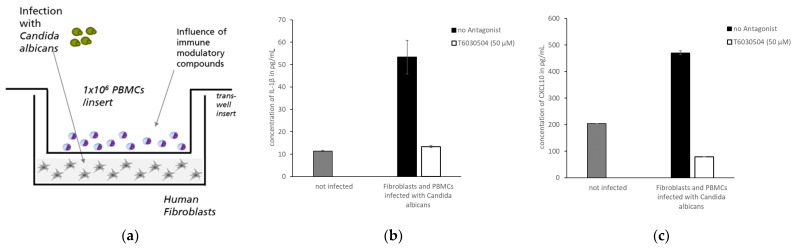
In vitro co-culture assay with human fibroblasts and PBMCs as infection model. (**a**) Schematic representation of the model: a confluent lawn of fibroblasts is grown on the bottom of a 24 well plate. PBMCs are placed in a membrane-sealed insert with a pore size of 3 µm above the confluent cell monolayer. The fibroblast cell layer is infected with *C. albicans* and the supernatant is collected after 48 h. (**b**) The secretion of IL-1β and CXCL10 in infected co-culture assay was measured in the presence or absence of T6030504. The PBMCs were pre-incubated (6 h) with 50 μM of T6030504. T6030504 pre-incubation results in significantly reduced cytokine levels of both IL-1β (**b**) and CXCL-10 (**c**) in the supernatant of the co-culture assay. The results are given for one representative duplicate experiment and expressed as means ± SD.

We previously showed that immune cells from human blood, like PBMCs and CD4^+^-T cells, activate dermal fibroblasts in 3D-skin infection models to engage in anti-microbial defense [18]. We hypothesized that a simplified version of a fibroblast-immune cell co-culture system would yield similar results, however, with much easier handling and reduced turnaround time. Cultivation of dermal fibroblast in a well-plate is less time consuming than setting up a skin model, as shown below. As a readout, the secretion of the antimicrobial peptides and chemokines by the fibroblasts to fend off intruding pathogens can be monitored in the media using standard ELISA assays. Figure 5a shows the general setup of the co-culture system. The dermal fibroblasts were cultivated in a 24-well plate up to confluence. Then the PBMCs were added to the well using a membrane-sealed trans-well insert prohibiting cellular transfer. Thus, the membrane physically separates PBMCs and dermal fibroblasts in two compartments, which can be incubated separately with pathogens and test-compounds. Using this co-culture system, we analyzed if T6030504 has an effect on the IL1-β and CXCL-1 secretion of dermal fibroblasts incubated with *C. albicans* in the presence of human PDMCs. Infection of the system was initiated by adding the fungal pathogen *C. albicans* to the fibroblast compartment. PBMCs were pre-incubated with T6030504 or mock treated as described in Materials and Methods. As shown in Figure 5b,c, the infection induces secretion of IL1-β and CXCL-1 into the medium supernatant. Pre-incubation of the PBMCs with T6030504 reduces the levels of both IL1-β and CXCL-1 secretion to the level of the not-infected system. Thus, T6030504 is able to suppress inflammatory reactions in a co-culture system with human PBMCs and fibroblasts in the presence of the fungal pathogen *C. albicans*.

#### 3.2.4. *C. albicans* Invasion into Immune-Responsive Skin Infection Model Is Reinstalled by T6030504

**Figure 6:**
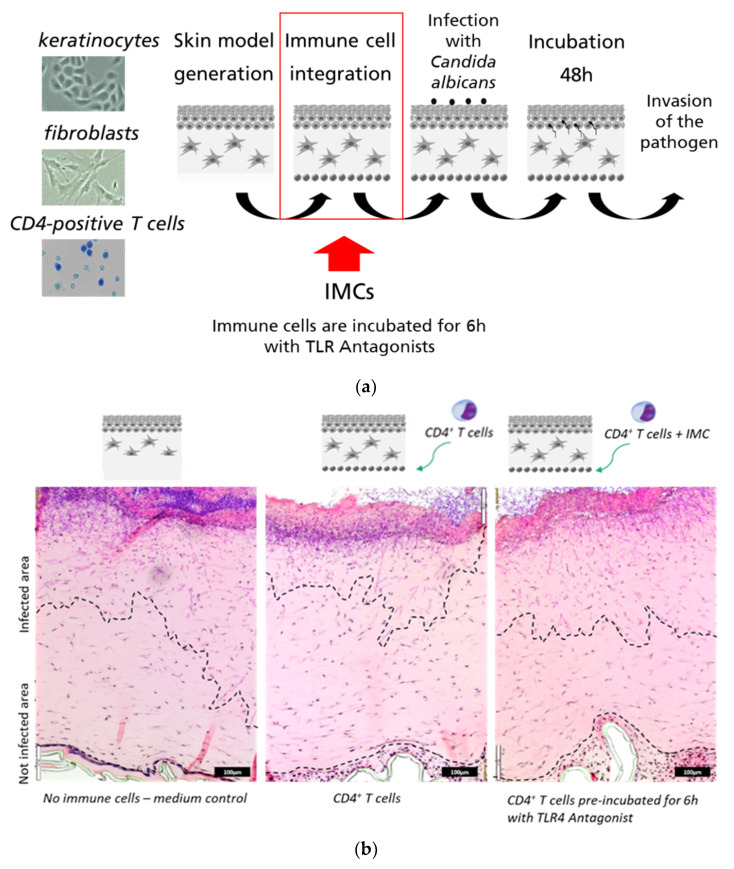

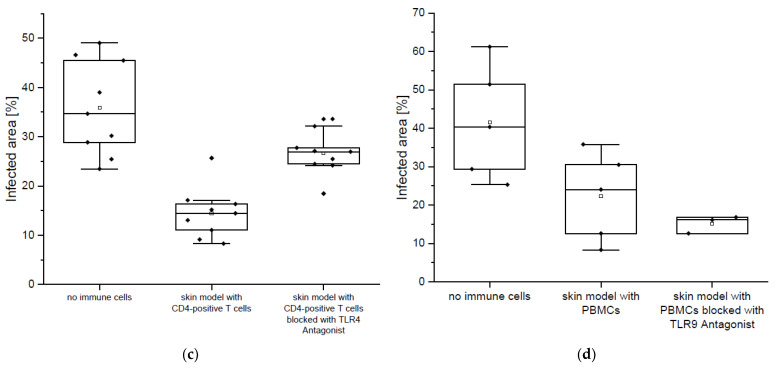
In vitro skin infection models supplemented with immune cells as infection model. (**a**) Schematic overview of immune-responsive skin models. Infection with *C. albicans* was performed on top of the epidermis, 24 h after immune cell integration (red frame). After 48 h of infection, the skin models were fixed and processed further. (**b**) H&E and PAS staining of *C. albicans* invasion into the skin models in the presence and absence of immune cells and antagonist T6030504. In the left picture the immune cells were pre-incubated for 6 h with the TLR4 antagonist T6030504 (50 µM). Black bars represents scale bar with 100 μm length (**c**) Dermal invasion was measured by determination of the percentage of infected area of the overall area from representative slices per skin model. The results shown in (**c**) represent means ± SD of three independent experiments (infection models), including three sections from each infection model. The invasion of *C. albicans* is significantly induced after the addition of the TLR4 antagonist to the CD4^+^ T cells. (**d**) The experiment was conducted as described in Figure 6**a**–**c**, except that the immune cells (PBMCs) were pre-incubated for 6 h with the previously published TLR9 Antagonist T5669070 (IC_50_ = 53.4 nM, CAS 920735-13-3) at a concentration of 1 µM. No significant increase in invasion of *C. albicans* was detected in the presence or absence of T5669070. Dermal invasion was evaluated by determination of the overall area and the infected area of one representative slice per skin model. The results are expressed as means ± SD of two independent experiments (one experiment includes 2–3 skin models).

The immune-responsive 3D-skin infection model [18] was employed in this study as the test system of highest complexity. This model is composed of three layers: epidermal keratinocytes (Ker-CT-cells) forming a stratum corneum on top, dermal fibroblast embedded in a collagen matrix (immortalized SF1-fibroblasts), and primary immune cells (CD4^+^ T-cells or PBMCs) embedded in a collagen matrix below the dermal layer (Figure 6a). In previous work, we showed that the immune cells confer a significant protection of the model against microbial invasion [18]. In contrast to the co-culture system shown in Figure 5, this 3D-model contains a complex matrix structure and an additional cellular component, keratinocytes, generating a barrier function in a similar way as in natural skin. The 3D-infection model also enables visualization of the invasion processes by histological methods, as shown in Figure 6b. We tested if the TLR4 antagonist T6030504 has an impact on defense against *C. albicans* in the immune-responsive 3D skin model. For this purpose, the isolated CD4^+^T cells were incubated with T6030504 or mock treated for 6 h before addition to the 3D-skin infection model. Figure 6b,c shows that T6030504 significantly inhibits the potential of the CD4^+^ T cells in the immune-competent skin model to restrict *C. albicans* invasion (Figure 6b,c). We also performed this experiment using a TLR9 inhibitor (CAS 920735-13-3, IC_50_ = 53.4 nM) published previously by us [19]. In this case, no difference was observed with regard to invasion of *C. albicans* into the 3D-model, in the presence or absence of the TLR9 antagonist (Figure 6d). This result further supports the role of T6030504 as inhibitor of TLR4-mediated immune response in the immune responsive 3D-tissue models developed.

## 4. Discussion

To combat infectious diseases especially with regard to rising antimicrobial resistance, it is essential to explore new paradigms for anti-infective therapy. Innate immunity is one of the first lines of immune defense, preventing pathogen invasion. Modulating innate immunity, therefore, might be a promising approach to support anti-infective therapy [2]. In this study, we identified novel TLR4 antagonists using a combination of computational drug discovery methods, including pharmacophore models and docking. To evaluate these molecules, we refrained from animal models, as they have been shown to be of limited reliability especially validating immune-modulating therapeutics of human. Instead, we developed a set of novel in vitro assays of human background with increasing complexity by adaptation of previously established immune-responsive infection models [17].

We identified 46 novel TLR4/MD2 antagonists in silico using a combination of pharmacophore modeling and docking as outlined in Figure 1. For one of the most promising compound in cell-based assays, T6030504, the putative docking site is shown in Figure 2. Interestingly several of the interactions predicted for T6030504 docked in the TLR4-hybrid MD-2 pocket have also been assigned for interaction with Eritoran, including Ile-117, Ser-118, and Phe-121.

Using TLR4-specific cell-based assays, we screened all 46 compounds in vitro for specificity and potency. The cell-based assays employed have been shown to be highly specific and sensitive for the analysis of single TLRs or heterodimers [26] and have been used previously to identify TLR4 agonists [14] and TLR9 antagonists [19]. Among the 46 molecules analyzed in vitro, we could identify seven novel compounds with activities close to 200 μM or below (see Supplemental Material). Two molecules, T6030504 and T6432438 showed the highest antagonistic activity both against the TLR4/MD2 and TLR4/CD14 receptor complex (Figure 2 and Supplemental Material). Due to the better availability of T6030504, this molecule was analyzed in more detail. The specificity for the TLR receptor complexes was confirmed using several additional cell-based assays specifically addressing other TLRs, including TLR7/8/9 and TLR1/2/5/6. None of them did show any effect on TLR-signaling (not shown). This indicates a highly specific interaction with the TLR4 co-receptor complexes and not with other elements of the signaling cascade, which is crucial to avoiding unwanted side effects. Importantly, the molecule did not show toxicity in the cell-based assays employed (see Figure 3). T6030504 was analyzed further in several assays involving primary immune cells (PBMCs), whole blood, and complex immune-responsive 3D-skin infection models, including CD4^+^ T cells. As shown in Figure 4, T6030504 was able to reduce IL1-β response in whole blood significantly. The difference observed in IL1-β response between isolated PBMCs and whole blood might be due to the difference in their origins. The PBMC assay shown in Figure 4a was performed using buffy coat from the blood donation center, reflecting a mixture of multiple donors, whereas the whole blood samples (Figure 4b) and the PBMCs for the more complex infection models (Figure 5 and Figure 6) were derived from individual donors. We could confirm antagonistic activity of T6030504 in co-culture experiments using PBMCs from individual donors, and primary human fibroblasts (Figure 5), as monitored by secretion of IL1-β and CXCL-10, two cytokines critical for innate immune response. Our results indicate that blocking of TLR4 signaling seems to be independent of the inducing ligand since both the cytokine inductions in response to LPS (Figure 4b) and to vital *C. albicans* (with O-Mannan as known TLR4-ligand [12]) (Figure 5) were strongly reduced by T6030504. Furthermore, an antagonistic effect of T6030504 in immune responsive 3D-skin infection models was observed by directly monitoring *C. albicans* invasion into the tissue. In this infection model, the protective effect of the CD4^+^ T cells on the 3D-tissue model was blocked after pre-incubation of the immune-cells with T6030504, resulting in increased invasion of *C. albicans* into the model (Figure 6). Previously, we could show that cell–cell communication between CD4^+^ T cells and dermal fibroblasts is critical for antimicrobial defense mechanisms in the dermis [18]. Addition of T6030504 to the immune cells resulted in increased invasion of *C. albicans* into the skin model, indicating that T6030504 is able to block TLR4 signaling in the immune cells and thereby disturbs cellular communication in the model. Addition of a known TLR9 antagonist [19] had no effect in this model (Figure 6d). Of note is that TLR4 has been shown previously to be critical for antifungal defense in an in vitro model of oral candidiasis based on the buccal epithelial carcinoma cell line TR146 and polymorphonuclear leukocytes (PMNs) [11]. This study is in line with our finding using the immune-responsive 3D-skin infection model and co-culture test systems combining primary immune cells with fibroblasts, further supporting a prominent role for TLR4 and its co-receptors in epithelial defense mechanisms. Previously, we could show that known inhibitors of TLR2 lead to a significantly increased microbial invasion into the infection model, due to a lack of activation of the antimicrobial defense mechanisms of derma fibroblasts [18]. Together with the results from this study, this further substantiates the observations that defense against fungal invasion in the skin is orchestrated in concert with several PRR receptors [12,29], and that this can be mimicked to a certain extend in vitro by combining several cell types in the appropriate environment. Several other immune receptors, including dectin and mannose receptors have been implicated as well in antifungal immune defense in several cell types, further enlarging this picture [30]. We expect that further development of human based 3D-tissue models will further reduce the dependencies on animals in drug development.

Our results indicate that T6030504 blocks TLR4 activity in the context of several different types of immune cells present in whole blood, including PBMCs and CD4^+^ T cells. Furthermore, the observation that the compound blocks TLR4 activation by LPS and *C. albicans*, indicates a ligand independent mechanism. The activity observed in whole blood furthermore indicates stability and availability of T6030504 for at least the period tested (more than 24 h at 37 °C), which is a prerequisite for further development into an anti-inflammatory drug. We could observe that the source of the immune cells is critical for the assays performed. This includes differences between individual donors and the use of pooled resources from blood donation centers. Furthermore, differences in the isolation process may be critical and affect the status of the various immune cell populations.

From the results in the past decades [31] it became evident, that a balance between protective inflammatory activity against microorganisms and deleterious overreaction of innate immunity has to be kept in balance. Especially activating innate immunity has been shown to be a double edged sword that needs to be controlled stringently, for example in sepsis [10]. Therefore, it will be necessary to provide both agonists and antagonist for the different indications targeted, in order to avoid deleterious effects for the patient, due to an imbalanced innate immunity induced by immune-modulators. This novel antagonist might contribute in the future to keep such a balance.

## Data Availability

Data is contained within the article or Supplementary Material.

## Supplementary Materials

The following supporting information can be downloaded at: https://www.mdpi.com/article/10.3390/microorganisms10020243/s1, Figure S1: Cell-based dose-response assays for validation of selected TLR4 Antagonist; Table S1: Validation of in silico identified potential TLR4-antagonists by cell-based assays.
