# Supplementary material for: Human-Based Immune Responsive In Vitro Infection Models for Validation of Novel TLR4 Antagonists Identified by Computational Discovery
Source: Microorganisms. 2022 Jan 22;10(2):243. doi: 10.3390/microorganisms10020243 (PMC8876567; doi:10.3390/microorganisms10020243)
Supplement: Supplementary file 1 [file microorganisms-10-00243-s001.zip › microorganisms-1563529-supplementary.pdf]

Supplemental material:

Table S1: Validation of in silico identified potential TLR4-antagonists by cell-based assays

Number	Compound ID	IC_{50} (TLR4/MD2) in µM
1	T6634389	1000
2	T6708618	n.d.
3	T6946416	1200
4	T6852075	n.d.
5	T6854457	n.d.
6	T7034365	3000*
**7**	**T6178967**	**203 (Fig. S1a)**
8	T6179020	2400*
9	T6416923	852
10	T6694846	n.d.
11	T7043595	697
12	T6535859	5000*
13	T5634443	n.d.
14	T5635346	7700*
15	T5655296	n.d.
16	T6634389	n.d.
17	T5874283	n.d.
18	T6630227	n.d.
**19**	**T6638124**	**135* (Fig. S1b)**
20	T6774706	n.d.
21	T7054880	n.d.
22	T0519-7352	281*
**23**	**T6890123**	**113* (Fig. S1c)**
24	T6896830	315*
25	T7052055	n.d.
26	T7076790	258*
27	T7086829	n.d.
28	T0501-2008	270*
29	T0506-1281	305*
30	T6549186	253*
**31**	**T6856838**	**144* (Fig. S1d)**
32	T6795578	n.d.
**33**	**T6763289**	**113***
34	T6946935	224*
35	T6724611	n.d.
36	T6698762	n.d.
37	T6696295	342*
38	T6684435	n.d.
**39**	**T6432438**	**56 (Fig. S1e)**
40	T6597001	n.d.
41	T6295054	262*
42	T6320987	295*
43	T6353036	492*
44	T6365740	286*
45	T6377118	241*
46	***T6030504***	***50 see (Fig. 2b)***

* IC_{50} values were interpolated based on the measured values using linear regression method. Data generated from the dose-response experiments for determination of IC_{50} of compounds in bold are shown in Fig. 1Sa-f. Data for compound #46 are shown in Fig. 3b (main part). N.d.: not detectable

Figure S1a

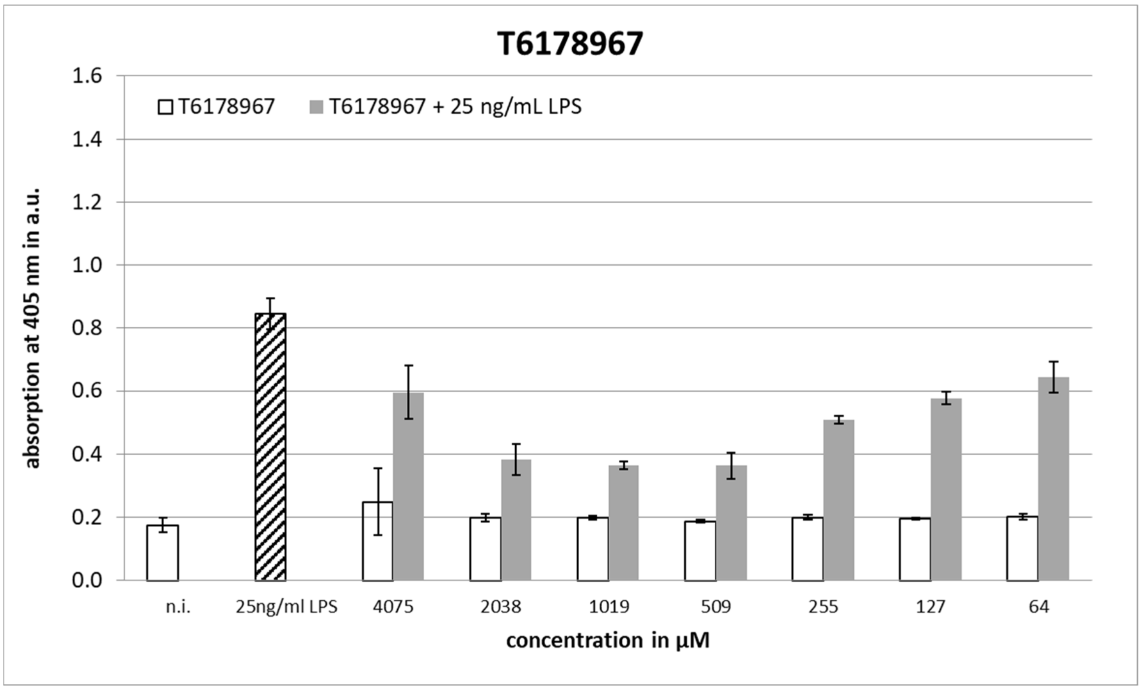

Figure S1b

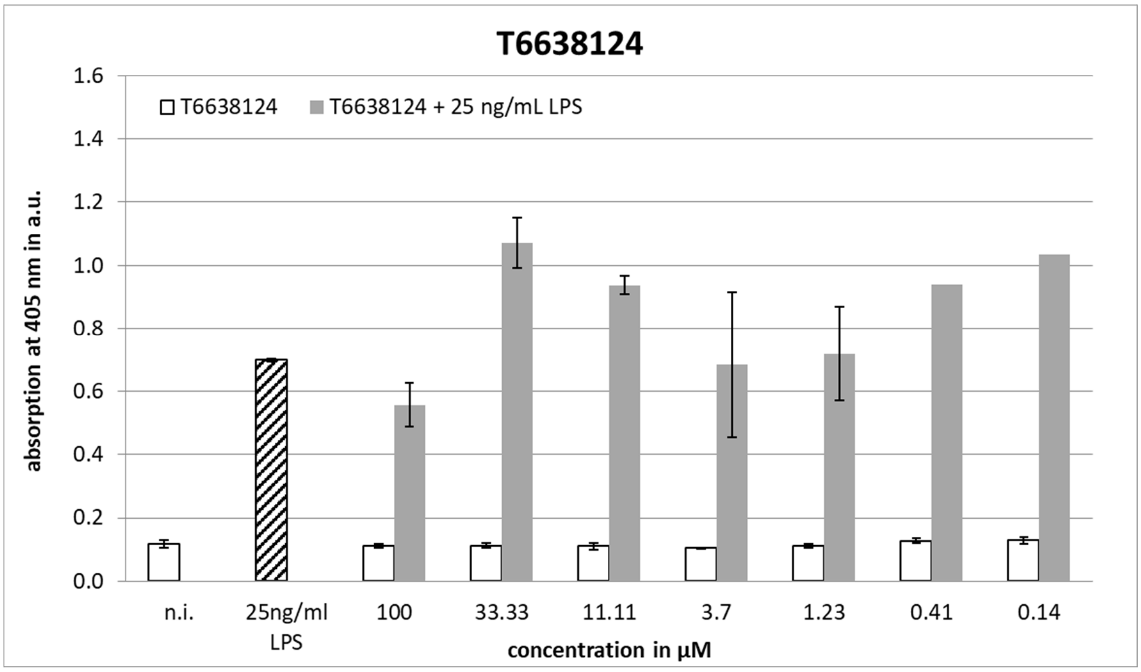

Figure S1c

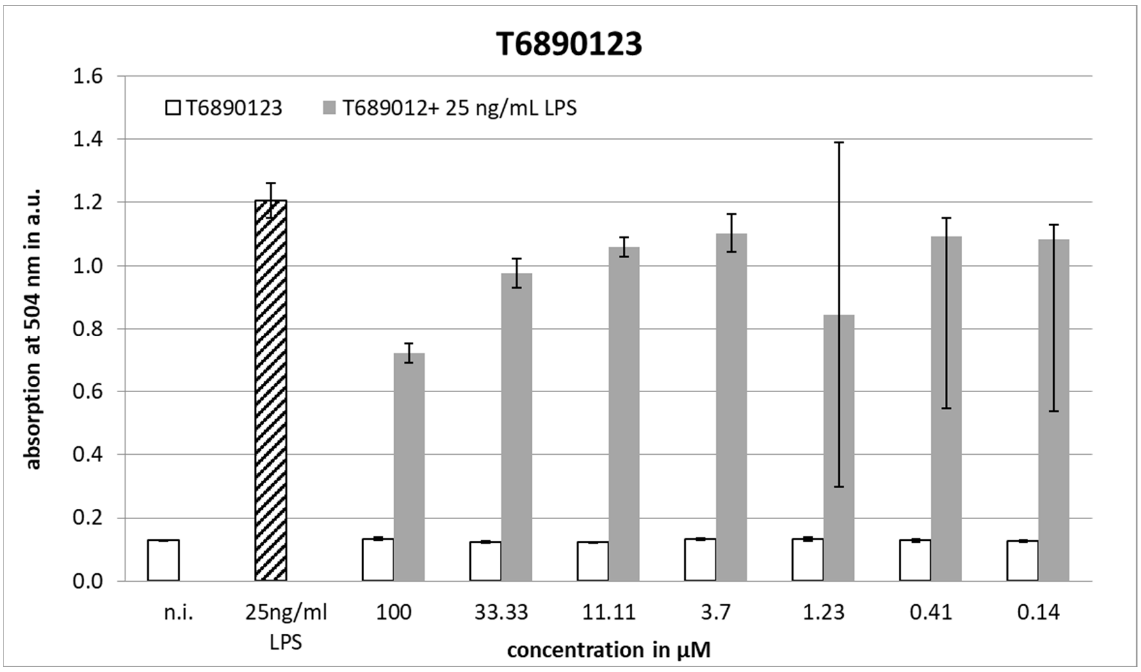

Figure S1d

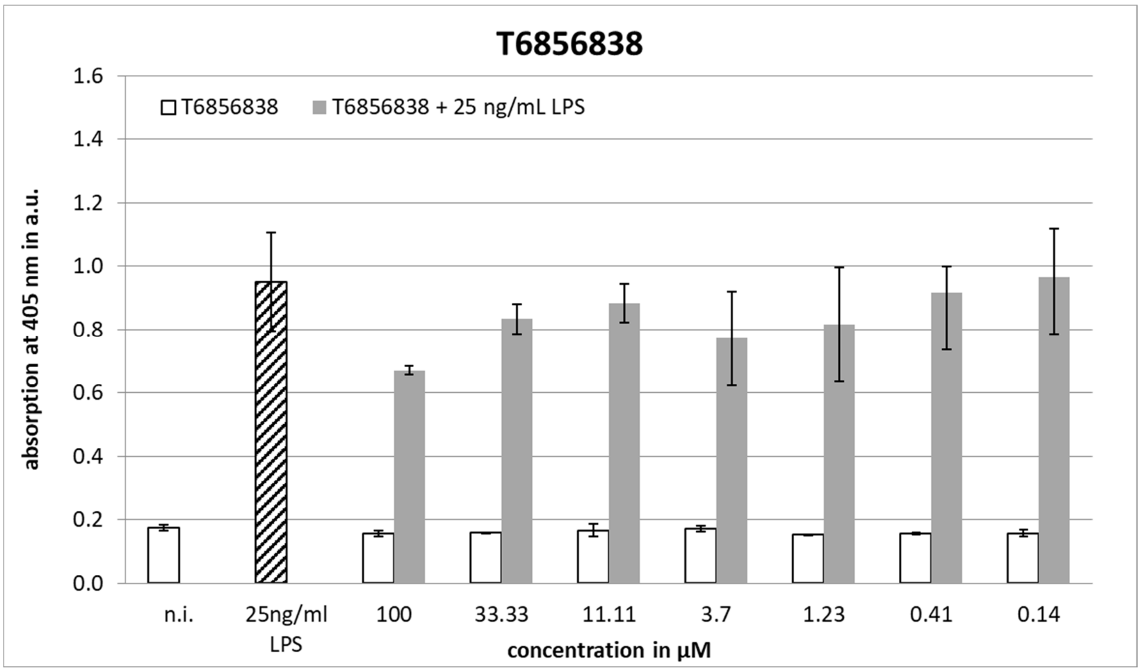

Figure S1e

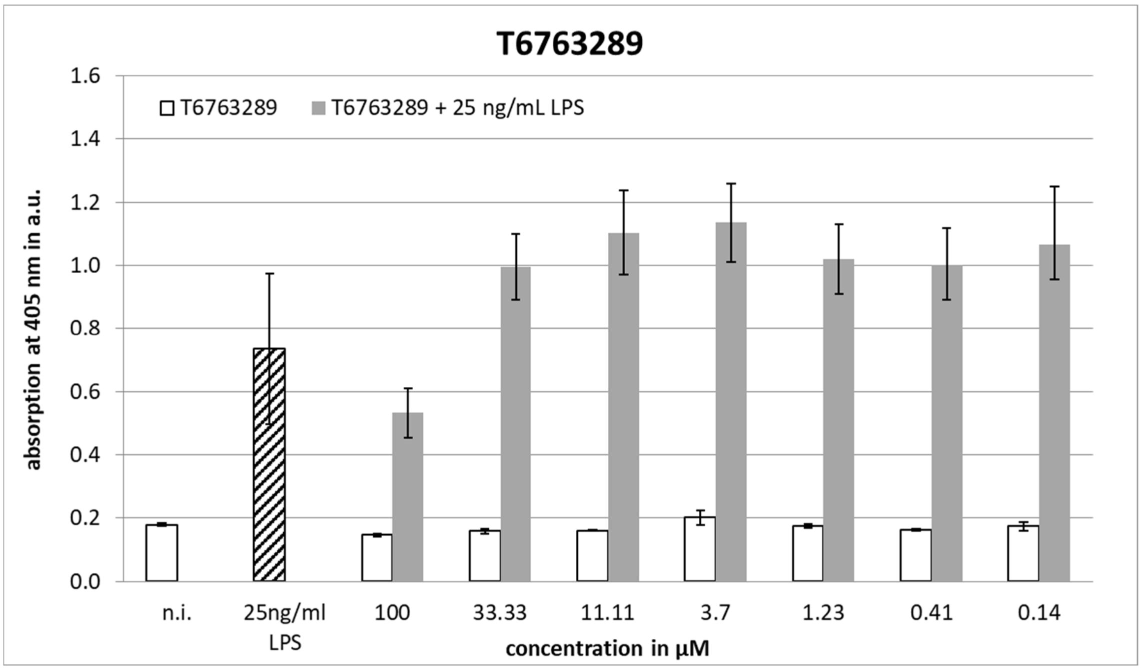

Figure S1f

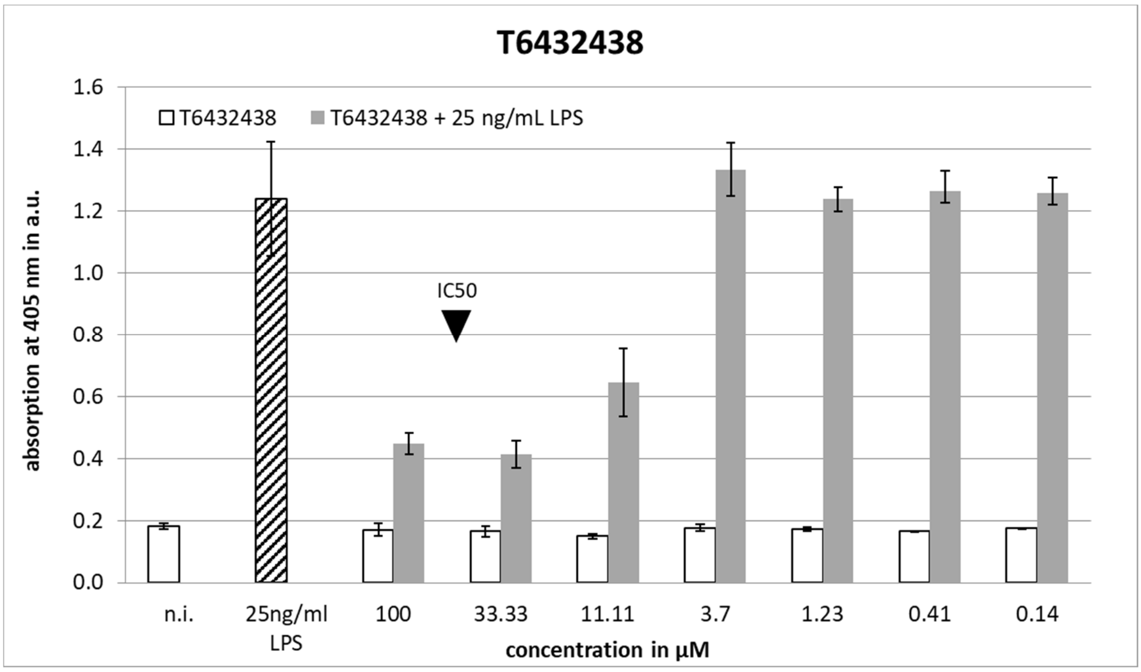

Figure S1. a–f Cell-based dose-response assays for selected TLR4 Antagonists: Dose-response analysis of the compounds indicated (T#, a-f) to identify the minimal inhibitory concentration necessary to block the TLR4 receptor complex in NIH 3T3 TLR4/MD2 reporter cell lines. LPS was used to activate the TLR4 receptor complex in NIH 3T3 TLR4/MD2 reporter cells with a concentration of 25 ng/ml. The supernatant was collected and the substrate p-NPP was added to monitor the NF-κB dependent SEAP secretion.
